# Upper metastable limit osmolality of urine as a predictor of kidney stone formation in children

**DOI:** 10.1007/s00240-018-1041-2

**Published:** 2018-01-22

**Authors:** Tadeusz Porowski, Jan K. Kirejczyk, Piotr Mrozek, Piotr Protas, Agata Kozerska, Łukasz Łabieniec, Krzysztof Szymański, Anna Wasilewska

**Affiliations:** 1 Department of Paediatric Nephrology, Children’s Clinical Hospital, Medical University of Bialystok, Bialystok, Poland; 2 Faculty of Health Sciences, Lomza State University of Applied Sciences, Lomza, Poland; 3 Faculty of Mechanical Engineering, Bialystok University of Technology, Bialystok, Poland; 4 Faculty of Physics, University of Bialystok, Bialystok, Poland

**Keywords:** Children, Urolithiasis supersaturation, Urinary osmolality, Water intake, Risk factors

## Abstract

High fluid intake has been universally recommended for kidney stone prophylaxis. We evaluated 24-h urine osmolality regarded as the best biomarker of optimal hydration and upper metastable limit osmolality after water evaporation from urine sample to the onset of spontaneous crystallization and its usefulness as a new risk index that would describe an individual lithogenic potential. We collected 24-h urine from 257 pediatric patients with kidney stones and 270 controls. After volume and osmolality assessment, the urine samples were subjected to volume reduction in vacuum rotavapor continued to the onset of an induced urinary crystallization. The upper metastable limit osmolality of urine sample was calculated based on its initial osmolality value and the amount of water reduction. Pediatric stone formers presented with higher urine volume and lower urine osmolality than healthy controls. Despite that, their urine samples required much lower volume reduction to induce the spontaneous crystallization than those of controls. The ROC analysis revealed an AUC for the upper metastable limit osmolality of 0.9300 (95% CI 0.9104–0.9496) for distinguishing between stone formers and healthy subjects. At the cutoff of 2696 mOsm/kg, the test provided sensitivity and specificity of 0.8638 and 0.8189, respectively. 24-h urine osmolality provided the information about current hydration status, whereas evaporation test estimated the urinary potential to crystalize dependent on urine composition. Upper metastable limit osmolality may estimate the individual lithogenic capability and identify people at risk to stone formation when exposed to dehydration.

## Introduction

Low urine volume and a high urine concentration are regarded as important kidney stone risk factors. Thus, high fluid intake has been universally recommended for kidney stone prophylaxis to produce more dilute urine, prevent supersaturation and crystallization of lithogenous salts [1]. Several studies have confirmed urine dilution as one of the most effective preventive measures for stone recurrence [2, 3]. Recent guidelines from American Urological Association recommended sufficient fluid intake to maintain urine volume in adults of 2.0–2.5 L [4]. In children, diuresis above 1 ml/kg per h, almost eliminated the risk of supersaturation for calcium oxalate, calcium phosphate and uric acid, thus protecting from the formation of the corresponding kidney stones [5].

24-h urine osmolality is regarded as the best biomarker of optimal hydration with respect to the risk of urolithiasis [6–8]. Urine osmolality depends on fluid intake but also on body size, diet-related osmotic load, physical activity, and other factors such as climate, environment and disease. Physiological values of plasma osmolality take precedence over urine concentration and are maintain within a narrow range despite different volumes of fluid intake. Small rise in plasma osmolality triggers a release of the hormone vasopressin, which in turn enhances water reabsorption along the collecting duct in the kidney and increase urine concentration. Therefore, urinary volume, and more specifically, urine osmolality are the end results reflecting the antidiuretic activity necessary to preserve water balance in human body [9]. Urine osmolality is an index of the concentration of osmotically active particles, particularly sodium, chloride, urea, and potassium. Glucose, when abundant in urine, can also add significantly to its osmolality. In children and young adults consuming a typical affluent Western-type diet urea excretion contributes to about 40% and sodium chloride to about 35–44% of total renal solute excretion [8, 10]. A rise of sodium excretion increases the values of urinary calcium and osmolality [11].

Hallson and Rose [12] studied urinary crystals after evaporation of urine sample in a vacuum rotatory evaporator at 37 °C. They have found production of crystals of a structure similar to those, which occur naturally in urine. According to authors, the procedure of rapid urine evaporation at body temperature seems to be analogous to the changes in urine concentration taking place during passing through the renal tubules to the tips of collecting ducts at the papillae [12]. In the present study, we applied the similar experimental test of urinary sample volume reduction leading to increase of its osmolality. The evaporation was continued to the onset of an induced urinary crystallization. We have hypothesized that urine from subjects at risk of urolithiasis represents greater capability to spontaneous crystallization of specific salts and requires a lesser degree of water reduction. If this proves to be truth, the method could determine the probability of kidney stone formation and/or effectiveness of its prevention in individual person using his/her urine sample.

The aim of the study was to assess: (1) 24-h urine osmolality and its relation to sex, age, weight, BMI z-score and urine volume; (2) upper metastable limit osmolality (UML_Osm_) after water evaporation from urine sample to the onset of spontaneous crystallization and its usefulness as a new universal risk index that would describe an individual potential to kidney stone formation, based on studied urine samples in children and adolescents with urolithiasis and their healthy counterparts.

## Patients and methods

The study was undertaken in the Department of Pediatric Nephrology, Medical University of Bialystok, Children’s Clinical Hospital, Poland. The study group consisted of 257 children and adolescents, aged 3–18 (median 13.59) years, diagnosed with urinary stones of different compositions, in which a 24-h urine was collected for initial metabolic evaluation. The control group comprised 270 age- and sex-matched healthy counterparts with negative family history and ultrasound imaging of kidney stones.

A single 24-h urine collection was provided at home while participants continued their customary diet. After voiding, urine was stored at 4 °C to maintain original conditions without addition of preservatives, and all measurements were conducted in hospital laboratory within 4 h of the end of the collection period. After assessment of urine volume, the initial osmolality of urine (IU_Osm_) was measured with a freezing point osmometer (model OS 3000; Marcel S.A., Poland).

**Fig. 1:**
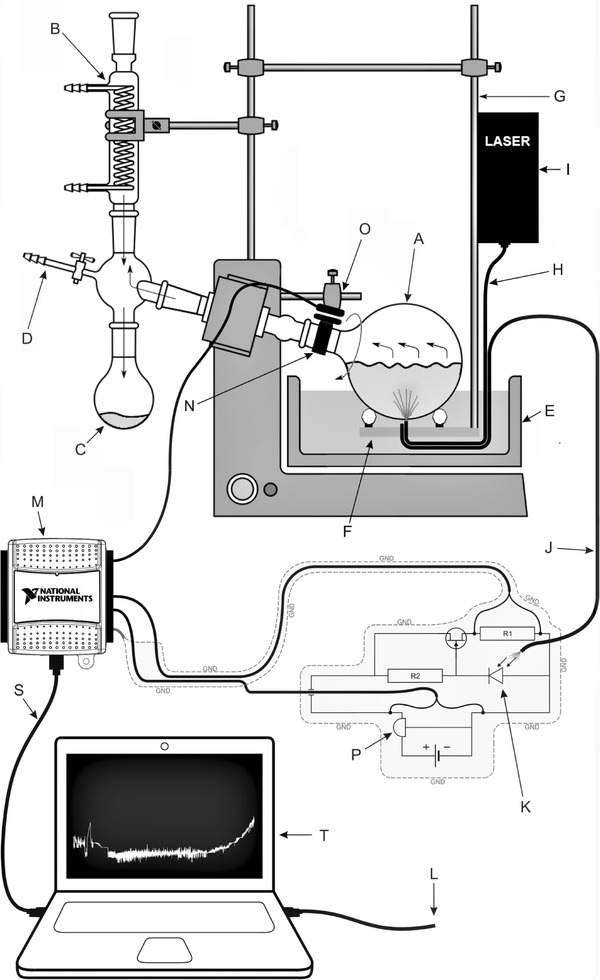
Setup for urine sample evaporation and turbidity measurements. *A* urine sample, *B* cooler, *C* container for distillate, *D* under pressure pump, *E* water bath, *F* sliding elements and holder of the waveguides, *G* elastic holder, *H* optical waveguide inserting the beam light, *I* laser, *J* optical waveguide for reading of scattered light, *K* PIN silicon photodiode, *M* data acquisition interface, *N* phase sensitive trigger, *O* sensor of the trigger, *P* voltage stabilizer, *S* USB cable, *L* automatic stop cable, *T* display

**Fig. 2:**
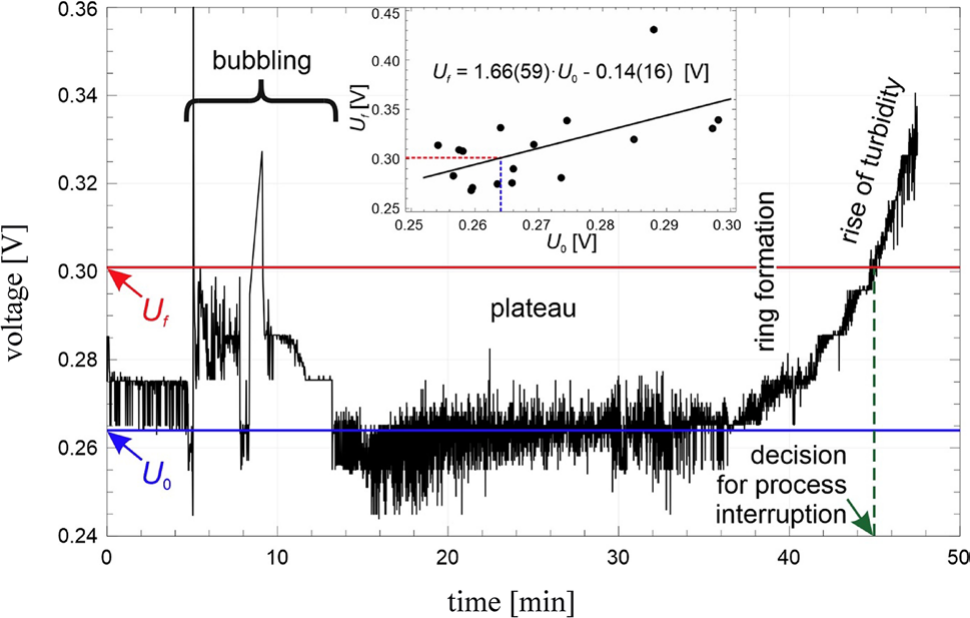
Recorded voltage (related to the scattered light as a measure of turbidity) vs. time for the process of evaporation of typical sample of urine. Decision to interrupt the evaporation was made automatically based on increase in urine sample turbidity and the recorded voltage. *U*_*f*_*inset* a correlation between the voltage of plateau *U*_0_ and the voltage related to the interruption moment *U*_f_

The unprepared urine samples (not filtrated, not centrifuged and at natural pH) of 50 ml were taken from 24-h urine collections, weighed on a precision scale and placed into a glass flask attached to the vacuum evaporator with under pressure of 3 hPa. The urine samples were maintained at 37 °C by moving glass flask down into the water bath and subjected to a rotational movement of 100 rpm. The distilled water was collected into glass cylinder. After a few minutes of evaporation, a bubbling of urine sample is observed which spontaneously disappear and subsequently urine sample remains visually clear for the next dozen of minutes. At certain moment, a dark ring of sediment, close to the glass wall, is formed on the top surface of the still transparent urine sample. Formation of the ring is the indication that fast precipitation inside urinary sample will occur soon. The measurement of urinary sample turbidity was performed by reflection geometry (Fig. 1). The system consisted of 660 nm red light laser source with a beam introduced to the sample by optical fiber. Another fiber transmit reflected light to the PIN silicon diode polarized in reverse direction realizing proportionality between the light intensity and the photocurrent. The measured photocurrent was recorded by personal computer and displayed on the screen. To increase signal to noise ratio, a trigger was used for phase sensitive detection during the rotation of the sample container. The optic fibers supplying the beam and receiving the backscattered light are mounted perpendicularly to the glass wall of the sample container, everything in a distilled water environment, to minimize the light refraction and reflection on the borders of media with different optical densities, i.e., water-glass-urine sample. A typical example of the recorded signal is shown in Fig. 2. There is a clearly seen phenomenon of gas bubbling, plateau of voltage, abbreviated by *U*_0_ and a rise in urine sample clouding. To calibrate the apparatus, a moment of beginning of urinary crystallization, i.e., fast increase in the sample’ turbidity was established at *U*_f_ (Fig. 2). The dispersion of the points is expected depending on the different properties of urine samples of investigated patients, however, a clear correlation between voltage of plateau *U*_0_ and the voltage related to the moment of interruption *U*_f_ was found as *U*_f_ = 1.66*U*_0_ − 0.14V (shown by solid line of inset in Fig. 2). In this way, a fully automatic process of turbidity recording and interruption of evaporation at the required level of turbidity was established.

Immediately after interruption, the weight of the remaining concentrated urine sample was measured and recorded. The weight reduction index (WR_Ind_) of the tested urine sample was expressed as the ratio of the initial weight of the sample to its weight at the end of water evaporation. The upper metastable limit osmolality (UML_Osm_) of studied urine sample at the onset of induced crystallization was calculated based on its initial osmolality value and the amount of water reduction (UML_Osm_ = IU_Osm_ × WR_Ind_).

The exclusion criteria comprised abnormalities in dipstick urinalysis (Bayer Diagnostic, Bridgend, UK), any infections or medications and inadequate 24-h urine collection assessed with urine creatinine excretion according to Remer et al. [13].

The protocol of study was approved by the ethics committee of the Medical University of Bialystok, Poland. Informed consent was obtained from the parents of all participants and from adolescents older than 16 years of age.

Statistical analyses were performed using Statistica®, ver. 10.0 PL (StatSoft Inc, Tulsa, OK). The Mann–Whitney U test was used for comparisons between two independent parameters, and the correlations were made with Spearman test. *p* values < 0.05 were considered to be statistically significant. R values of correlation coefficients ≤ 0.35 were considered to represent weak a relationship, 0.36–0.67 moderate association and 0.68–1.0 strong correlation [14]. Additionally, ROC curve was computed for UML_Osm_ to evaluate the predictive capacity of the evaporation test and obtain the value that most reliably differentiated between stone formers and healthy subjects.

## Results

**Fig. 3:**
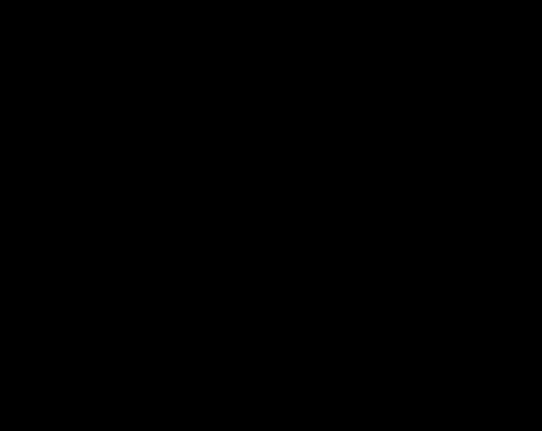
Effect of age (**a**) weight (**b**), BMI *z*-score (**c**), and urine volume (**d**) on 24-h urine osmolality (IU_Osm_). A regression lines, Spearman’s rank correlation coefficients and *p* values are shown for stone formers and healthy controls

The comparisons of the demographics, urine volumes, IU_Osm_, UML_Osm_ and the WR_Ind_ between the stone formers and the controls are summarized in Table 1. The median values of 24-h total urine volume as well as urine volume calculated per body weight were significantly higher in the patients when compared to controls (1225 vs 911 ml/24 h, *p* < 0.001 and 31.37 vs 23.17 ml/kg/24 h, *p* < 0.001, respectively). Males revealed significantly higher medians of IU_Osm_ comparing to females in study group and controls: 460 vs 392 mOsm/kg, *p* = 0.014 and 632 vs 515 mOsm/kg, *p* = 0.010, respectively. Nevertheless, the both genders have shown the same trends of examined relationships and their proportions were the same in the study and control groups, thus, they were further analyzed together. 24-h IU_Osm_ was significantly lower in stone formers comparing to their healthy counterparts (422 vs 579 mOsm/kg, *p* < 0.001). The associations between the IU_Osm_ and age, weight, BMI z-score and urine volume are presented in Fig. 3. Across the study age spectrum, 24-h IU_Osm_ presented weak positive relationships with age, weight and BMI z-score (panel A, B, and C, respectively) and strong negative correlation with urine volume in both groups (panel D).

**Table 1:** The values of studied parameters in pediatric stone formers and healthy controls

Characteristics	Stone formers	Healthy controls	*p*
*N*	257 (♂ 131, ♀ 126)	270 (♂ 138, ♀ 132)	
Age (years)	13.59 (3.75–17.94)	11.50 (4.31–17.83)	0.1
Weight (kg)	44.00 (13.50–75.00)	44.40 (16.90–89.00)	0.12
Height (cm)	155.00 (93.00–180.00)	150.00 (108.5–181.0)	0.82
BMI z-score	0.13 (− 1.61–1.54)	0.37 (− 1.52–2.31)	0.18
Creatinine (mg/kg/24 h)	19.39 (19.10–31.52)	17.90 (11.00–27.29)	0.000
Urine volume (ml/24 h)	1225 (409–3000)	911 (337–2200)	0.000
Urine volume (ml/kg/24 h)	31.37 (15.11–68.96)	23.17 (6.69–50.66)	0.000
Osmolar load (mOsm/24 h)	548.00 (232.30–1135.20)	484.82 (247.50–1057.92)	0.000
Initial 24-h urine osmolality (IU_Osm_) (mOsm/kg H_2_O)	422.00 (227.00–853.00)	579.00 (256.00–1069.00)	0.000
Upper metastable limit osmolality (UML_Osm_) (mOsm/kg H_2_O)	2044.64 (1007.03–2955.48)	3677.11 (2289.22–5855.62)	0.000
Weight reduction index (WR_Ind_) (g/g)	4.42 (2.58–9.57)	7.21 (2.74–15.31)	0.000

Values are presented as median, with the range (5–95%) given in parenthesis

**Fig. 4:**
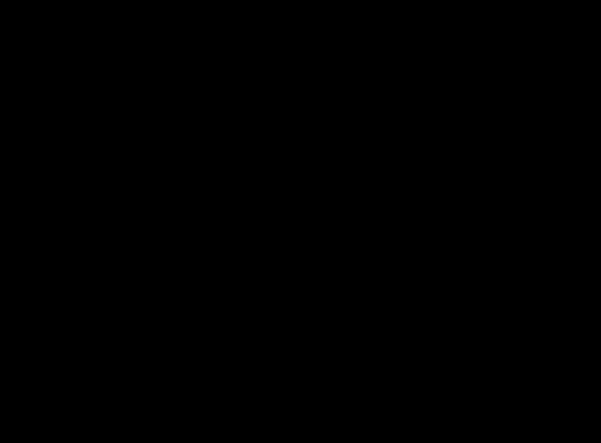
Weight reduction index (WR_Ind_) obtained during evaporation test of urinary samples plotted against initial values of urine osmolality (IU_Osm_). A regression line, Spearman’s rank correlation coefficients and p values are shown for stone formers and healthy controls

**Fig. 5:**
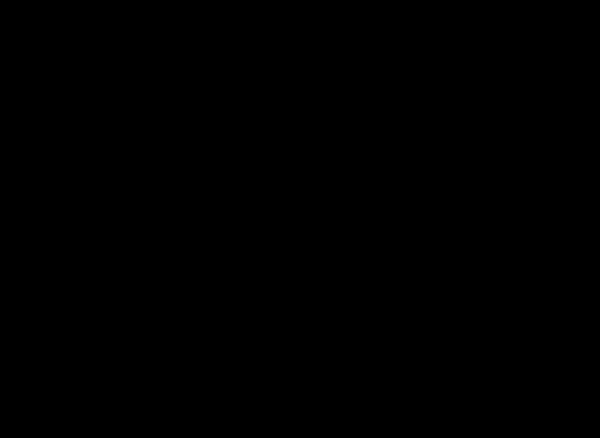
Upper metastable limit osmolality (UML_Osm_) obtained at the onset of induced urine crystallization plotted against initial values of urine osmolality (IU_Osm_). A regression lines, Spearman’s rank correlation coefficients and p values are shown for stone formers and healthy controls

**Fig. 6:**
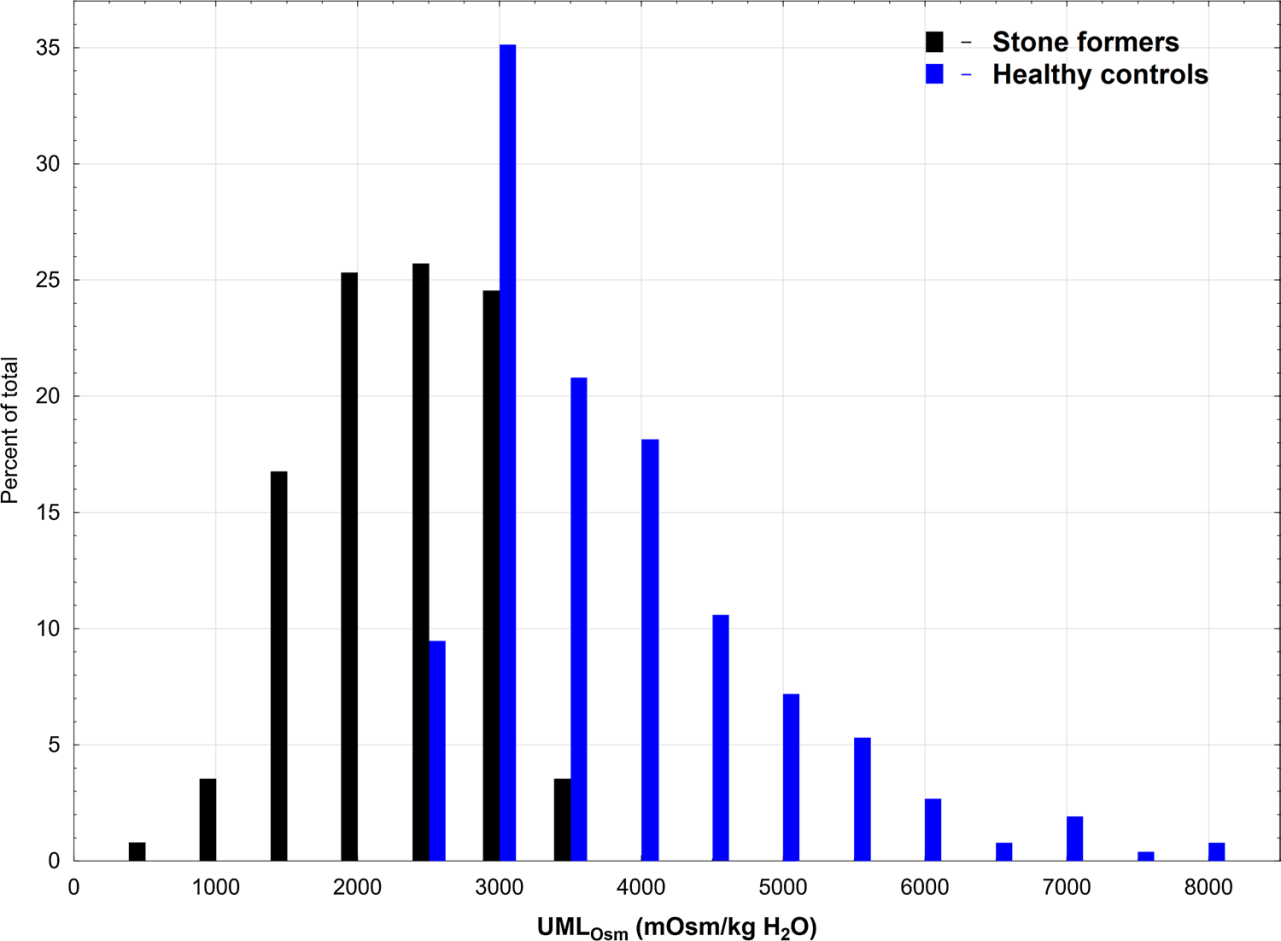
Frequency distribution of upper metastable limit osmolality (UML_Osm_) of urine in pediatric kidney stone formers and healthy controls

**Fig. 7:**
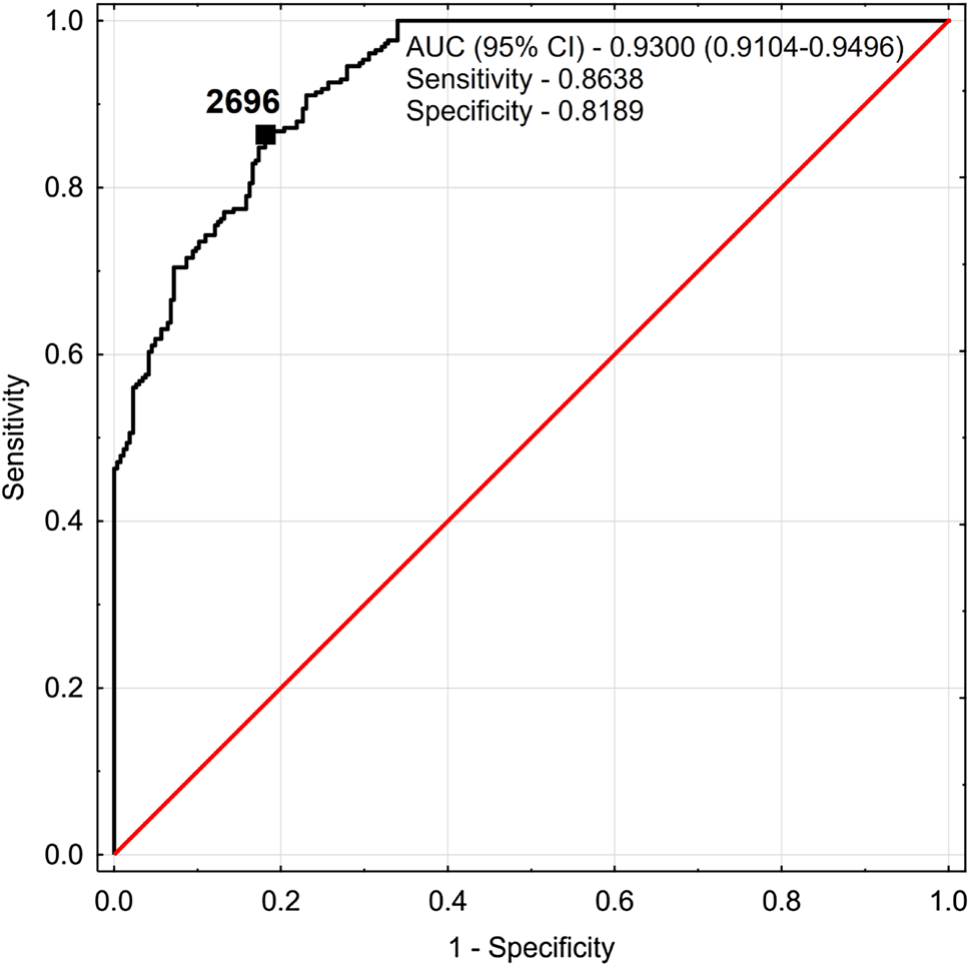
Receiver operator characteristic curve calculated for upper metastable limit osmolality (UML_Osm_) of urine determined from our comparison of pediatric stone formers and healthy controls and based on cutoff value of 2696 mOsm/kg H_2_O

The median of WR_Ind_ was significantly lower in urolithiasis group when compared to controls: 4.42 vs 7.21 g/g, (*p* < 0.0001) and showed strong negative relationship with IU_Osm_ in urolithiasis and control groups (*r* = − 0.689 and *r* = − 0.820, respectively) (Fig. 4). The median of UML_Osm_ in nephrolithiasis patients was significantly lower than that of healthy individuals: 2044.64 and 3677.11 mOsm/kg, respectively (*p* < 0.001). Values of UML_Osm_ revealed weak positive association with IU_Osm_ in patients (*R* = 0.282; *p* < 0.05), whereas did not present such relationship in controls (*R* = − 0.009, *p* > 0.05) (Fig. 5). The analysis of the frequency distribution of UML_Osm_ showed shift of kidney stone formers to lower values and healthy controls to higher values of osmolality (Fig. 6). However, differences in UML_Osm_ between the groups were not clear-cut, with overlap of both groups in range of 2289–2955 mOsm/kg (5th and 95th percentile for controls and stone formers, respectively). Based on ROC analysis, the urine sample evaporation test with calculation of UML_Osm_ revealed high diagnostic performance to discriminate subjects at risk of urolithiasis with AUC of 0.9300 (95% CI 0.9104–0.9496). The highest accuracy cutoff value of UML_Osm_ selected at 2696 mOsm/kg, provided sensitivity and specificity of 0.8638 and 0.8189, respectively (Fig. 7).

## Discussion

Large personal variability in water requirements exists due to different body sizes, diet-related osmotic load, physical activity and environment. Measurement of 24-h urine volume and its osmolality may help patients and/or health-care professionals to quantify individual 24-h hydration status with respect to lithogenic risk. In industrialized countries, a 24-h urine osmolality > 830 mOsm/kg was regarded as criterion for dehydration in both adult and children [15]. However, growing evidence suggest that above threshold for hypohydration is not optimal from health standpoint. Perrier and colleagues [6] established a 24-h urine osmolality ≤ 500 mOsm/kg as an indicator of optimal hydration, representing a total daily fluid intake adequate to compensate for daily losses, body water homeostasis and to ensure urinary output sufficient to reduce the risk of urolithiasis. Values of 24-h urine osmolality > 500 mOsm/kg were associated with higher than baseline plasma vasopressin concentrations suggestive of antidiuretic effort [6].

In the present study, the 24-h urinary osmotic excretion was greater and IU_Osm_ was lower in patients when compared to healthy subjects, which indicates the better hydration in stone formers (Table 1). Six percent of patients and 21% of controls presented with 24-h IU_Osm_ exceeding the established threshold for dehydration, whereas 35% of patients and 60% of controls overpassed the value of IU_Osm_ indicating optimal hydration from lithogenic standpoint. The difference in 24-h urine osmolality between groups may be explained by higher total 24-h urine volume in patients comparing to controls. Other researchers also observed higher urine output in children with nephrolithiasis and concomitant hypercalciuria comparing to healthy children [16]. In our earlier study, hypercalciuria coexisted in 60% of urolithiasis patients cohort [17]. We can expect that some natural mechanisms counteracting stone formation exist leading to increased thirst and urine volume. Despite the first stone episode, we could not exclude the possibility that some of patients had been advised elsewhere to increase their daily fluid intake for preventive reasons.

We observed the gender difference in IU_Osm_, with higher values in boys, a feature that was demonstrated in several industrialized countries before [18–20]. Across the study age spectrum, 24-h IU_Osm_ presented weak positive association with age. In other studies, IU_Osm_ increased during the second and third year of life up to the common level of children, adolescents and young adults followed by slow decrease after the age of 20 [8, 21].

In the second experimental part of our study, 24-h urine samples were brought to rapid water evaporation in strictly specified conditions. The urine samples were evaporated to the point of metastable limit for insoluble salts resulting in spontaneous crystallization [22]. Further evaporation leads to the formation of detectable crystals and clouding of the sample causing a decrease in the sample’s laser light transmissivity. We examined an unprepared 24-h urine samples at the original pH of urine containing all lithogenic and stone-inhibitory substances, without promoting any type of crystallization. The urinary samples of stone formers required 4.42 times, whereas those of healthy controls demanded 7.21 times of weight reduction to overcome an upper limit of metastability and bring about the obvious crystallization. The UML_Osm_ values became largely separated between groups with much lower values of urine osmolality in stone formers when compared to controls. Despite some overlap of UML_Osm_ values between the groups, the ROC analysis showed that evaporation test with estimation of UML_Osm_ could indicate individuals at risk for renal stone formation when exposed to dehydration. Therefore, the measurement of both IU_Osm_ and experimental UML_Osm_ provided the information about individual hydration status with respect to lithogenic risk and his/her urinary potential to crystalize dependent mainly on urine composition.

We are aware of some important limitations of this preliminary study. We did consider neither 24-h urinary biochemical profile nor stone composition of the study participants. Another important issue is that we did not take into account urinary pH and possible small pH changes during evaporation test what may influence solubility of precipitated salts. Finally, the evaporation test requires laboratory standardization to be used by other researchers.

## Conclusions

24-h urine osmolality was a good indicator of current hydration, however, did not directly reflect the individual risk of urolithiasis. Pediatric stone formers presented with higher 24-h urine volume and lower urine osmolality than healthy controls. Despite that urine samples of stone formers required much lower volume reduction to overcome an upper limit of metastability and induce the spontaneous crystallization of insoluble salts than those of healthy controls. Measuring the capability of urine to crystallize during water evaporation, i.e., upper metastable limit osmolality may estimate the individual lithogenic potential and identify people at risk to stone formation when exposed to dehydration.
